# Process evaluation of a brief messaging intervention to improve diabetes treatment adherence in sub-Saharan Africa

**DOI:** 10.1186/s12889-021-11552-8

**Published:** 2021-08-21

**Authors:** N. Leon, H. Namadingo, S. Cooper, K. Bobrow, C. Mwantisi, M. Nyasulu, N. Sicwebu, A. Crampin, N. Levitt, A. Farmer

**Affiliations:** 1 South African Medical Research Council, Fransie van Zyl Drive, 7535, Tygerberg, Cape Town, South Africa; 2 Department of Epidemiology, School of Public Health, Brown University, Providence, RI USA; 3 Malawi Epidemiology and Intervention Research Unit, Lilongwe, Malawi; 4 Chronic Disease Initiative for Africa, University of Cape Town, Cape Town, South Africa; 5 Division of Social and Behavioural Science, School of Family Medicine and Public Health, University of Cape Town, Cape Town, South Africa; 6 London School of Hygiene and Tropical Medicine, London, UK; 7 Nuffield Department of Primary Care Health Sciences, University of Oxford, Oxford, UK

**Keywords:** Qualitative process evaluation, Diabetes adherence, Randomised trial, Digital health, Brief text messaging, Sub-Saharan Africa, Low- and middle-income countries

## Abstract

**Background:**

The SMS text Adherence suppoRt for people with type 2 diabetes (StAR2D) intervention is a pragmatic randomised controlled trial, testing the effectiveness of brief text messaging for improving clinical outcomes and medication adherence. The intervention did not impact glycaemic control. We conducted a pre-and post-trial process evaluation alongside the StAR2D study in Malawi and South Africa, exploring the experiences and perceptions of patient participants, to better understand potential underlying reasons for the trial outcomes.

**Methods:**

We employed a qualitative research design, including conducting semi structured in-depth interviews and focus groups at both trial sites. Purposive sampling was used to ensure representation of a wide range of patients with type 2 diabetes with regards to age, gender, ethnicity, language, and duration of diabetes. We interviewed the same participants at baseline and at the end of the trial. We used within-case and across-case thematic analysis to identify key themes.

**Results:**

Brief messages delivered by text were acceptable and useful for addressing informational and support needs for participants. Some participants reported behaviour changes because of the text reminders and advice on a healthy lifestyle. Both participating in the trial and the messages were experienced as a source of support, caring, and motivation. Participants’ ability to act on the messages was limited. A common theme was frustration over the lack of ability to effectively control one’s blood glucose level. They reported a range of routinised, partial diabetes care adherence behaviours, shaped by complex and interacting individual, social, and health service factors. Participant responses and intervention impact were similar across sites, despite differences in health services.

**Conclusion:**

This process evaluation provided context and insight into the factors influencing participants’ engagement with the text messaging intervention. The complex context in which patients take their diabetes medication, may explain in part, why brief text messaging may have been insufficient to bring about changes in health outcomes. The scale of need for self-management and health service support, suggests that health system strengthening, and other forms of self-management support should accompany digital communication interventions. (Current Controlled Trials ISRCTN70768808, registered 03/08/2015.)

**Supplementary Information:**

The online version contains supplementary material available at 10.1186/s12889-021-11552-8.

## Background

### Introduction

Type 2 diabetes mellitus (T2DM) is a major global public health concern [1]. Low- and middle-income countries (LMICs) are disproportionally affected by a large and growing burden of premature morbidity and mortality associated with chronically elevated blood glucose levels [1, 2], with an estimated 75% of people with diabetes living in LMICs [3]. In sub-Saharan Africa, it was estimated that 12.1 million people were living with diabetes in 2010, and numbers are projected to increase to 23.9 million by 2030 [4]. T2DM is the most common form accounting for over 90% of cases [5]. Health outcomes for people treated for T2DM could be substantially improved in sub-Saharan Africa, but failure to take diabetes medicine regularly and to follow a healthy lifestyle (described as non-adherence), can result in failure to achieve the benefits of effective medical treatment and improved health outcomes [6, 7]. Medication non-adherence is common, estimated at 30–50% of people with long-term conditions, across diverse diseases and patient groups [8]. In sub-Saharan Africa, glucose levels are adequately controlled in less than a third (27%) of the diabetic patients, and 60% or more have complications [5, 9]. Reasons for not collecting or taking medications as intended are well documented and include psychological factors, lack of social support, low levels of health literacy, and health care service interactions that do not effectively support self-management [10–15]. Diabetes prevalence and associated morbidity, mortality, and health expenditure are placing a major social, financial and health service burden on the world [16], which is worsened in overburdened and low resource settings, such as sub-Saharan Africa.

Targeted digital client communication, using mobile phone-based, brief text messaging (also referred to as short message service or SMS), has been recommended for adherence support for a range of health issues and for smoking cessation and adherence to anti-retroviral treatment [17, 18]. Brief text messaging has also shown promise for supporting adherence for diabetes [19–24]. However, there are limits to the utility of the evidence for implementing digital brief messaging at scale, as results are often mixed or uncertain [17, 20, 21, 25–28], or come from smaller scale studies, and stakeholder views are not always explored [20, 21, 29–32]. Even where evidence from experimental studies (such as randomised controlled trials) are available, it is difficult to understand the underlying reasons for the effect (or lack thereof), as such studies do not illuminate the underlying pathways of behaviour change [21, 31]. Process evaluations alongside these studies, can generate insights into the potential underlying influences and causal pathways, and enhance the utility, and applicability of the evidence [12, 31, 33–36].

We conducted a pre-and post-trial process evaluation alongside a randomised controlled trial that tested the effectiveness of brief text messaging in improving health outcomes and medication adherence compared to usual care in patients with T2DM in two LMICs, Malawi (MLW) and South Africa (SA). The aim of the process evaluation was to explore the experiences and perceptions of patient participants, to help understand the potential underlying reasons for the trial outcomes. Understanding patient participants’ expectations, beliefs, responses, and satisfaction with the intervention is also important for assessing the acceptability and feasibility of the intervention for future upscaling [12, 31, 33, 34, 37].

### The trial study

SMS text Adherence suppoRt for people with type 2 diabetes (StAR2D) intervention is a pragmatic randomised controlled trial in two sub-Sharan settings, Malawi, and South Africa (Current Controlled Trials ISRCTN70768808, registered 03/08/2015). The trial tested the effectiveness of sending brief, automated SMS text-messages, using the patient’s own mobile phone, for improving clinical outcomes and adherence to refilling medicine, in patients with T2DM, compared to controls. The trial evaluated a range of clinical and behavioural outcomes. The primary trial outcome was glucose control (HbA1c), secondary clinical outcomes included control of blood pressure (systolic blood pressure) and measures of the proportions of the participants reaching treatment goals (with blood glucose, HbA1c < 8% and systolic blood pressure < 140 mmHg.) The main behavioural outcome was an objective measure of adherence; the proportion of days covered for prescribed medications.

Mobile messaging involved receiving up to four SMS text-messages each week, for 12 months, promoting regular medication refill, medication, and healthy lifestyle changes. Messages were delivered via a remote server, in one of four local languages (English, Chichewa, isiXhosa, and Afrikaans). Messages were one-directional and semi-tailored to reflect the clinic setting. Participants could choose their preferred language and timing of message delivery. Controls had usual care and received infrequent, trial-related messages (no more than 1 per month), that were sent to all participants. Formative research (that included input from patient and staff stakeholders) contributed an evidence and theory-informed set of brief text-messages and delivery mechanisms, as documented elsewhere [38]. The trial protocol provide further details of the study design and intervention mechanisms [39].

The trial was completed in October 2018, and publication of the results are in submission (see preliminary results in the ISRCTN Registry) [40]. To summarise, trial results did not provide convincing evidence that well-designed text messages lead to changes in glycemic control for people with T2DM at 12 months, though there was evidence of clinically important impact on blood pressure and overall cardiovascular risk. Findings were similar between trial sites (paper in submission). The process evaluation findings presented here offer some insights about the possible underlying reasons for the lack of positive effects on the primary outcome in this trial.

## Method

### Setting

The process evaluation study was conducted alongside the main trial at the same local healthcare facilities in Lilongwe, Malawi and Cape Town, South Africa. Both sites serve low-income communities and have a high burden of T2DM. The estimated prevalence of diabetes in Malawi, for 2014, was 6.6% for men and 6% for women. For South Africa, the estimated prevalence was higher for men at 9.7 and 12.6% for women [41]. Diabetes care is provided free of charge at both sites, including essential medicines and laboratory tests at no cost to patients.

In Lilongwe, patients with diabetes have scheduled appointments every 3 months for a health check and to refill their medications. In Cape Town, the medical check-ups are bi-annual, and the medication refills are once a month or every 2 months. The Lilongwe health facility serves patients from the city as well as those living in the surrounding areas (up to 2 h by public transport), whereas in Cape Town, most patients live within a short distance away (the furthest being a 30-min walk away).

### Study design

We carried out a process evaluation using qualitative research methods, in a phenomenological approach that aimed to examine the lived experiences of humans, through systematic collection and analysis of narrative material [42]. We collected data at both sites using semi structured in-depth interviews (IDIs) and focus groups discussions (FGDs) with purposive and convenience sampling of stakeholders. Combining IDIs and FGDs facilitate a deeper comparison of perspectives, improve data completeness and enhance the trustworthiness of findings [43]. While interviews enable one to explore individual views and experiences in detail, the conversational nature and interaction in a focus group can help generate additional insights, test and refine understandings from individual interviews, and stimulate commentary that may not have been elicited from individual interviews [44].

Participants recruited were men and women, 18 and older, clinically diagnosed with T2DM and taking diabetes treatment (oral medication or both oral and insulin). Purposive sampling was used to ensure representation of the wide range of patients with T2DM in the study areas, with regards to age, gender, ethnicity, language, and duration of diabetes. For the pre-trial interviews, we selected participants that met our criteria, from an anonymized list of participants that had been recruited for the trial. A research assistant, with the help of the trial staff, invited recruits to participate in a pre-trial individual interview, in person in waiting rooms or via telephone Pre-trial interviews were conducted after enrolment into the trial and before the participant was randomised. We interviewed the same participants at the end of the trial. We did not monitor the number of participants who declined to participate. Participants were reimbursed for their travel and time following local country research guidelines.

We conducted individual interviews with participants allocated to the intervention group, as well as those in the control group receiving usual care (See Table 1 for breakdown). Control group participant messaging were infrequent, mainly trial related messages. We wanted to better understand both intervention and control participations’ experiences, to see if and how their trial participation may have contributed to their responses. In a similar study on adherence to hypertension treatment, we found that control participants also valued their participation in the trial [12].

**Table 1:** Data sources and demographics of participants

Data sources	Lilongwe	Cape Town
**IDIs (Intervention group)**		
Matched pairs, *n* = 14 (28 IDIs) Post-trial only, *n* = 2 IDIs	Total = 8 4 Female participants	Total = 8 (6 matched, 2 post-trial only) 5 Female participants
	4 Male participants	3 Male participants
**IDIs (Control group)**		
Matched pairs, *n* = 12 (24 IDIs)	Total = 7 2 Female participants	Total = 5 3 Female participants
	5 Male participants	2 Male participants
**FGDs (Intervention group)**		
Post-trial FGD, *n* = 4 (25 participants)	Total = 2 (12 participants) 1 Female FGD, 6 participants	Total = 2 (13 participants) 1 Female FGD, 6 participants
	1 Male FGD, 6 participants	1 Male FGD, 7 participants

The process evaluation builds on the StAR2D formative work that was conducted to develop a evidence- and theory-informed brief text message intervention [45], using the Capability, Opportunity, Motivation - Behaviour (COM-B) behaviour change framework. The COM-B framework consolidates evidence from several other behaviour change theories and developed a behaviour change taxonomy that can account for contextual factors influencing behaviour (like socio-economic conditions and health service factors) [46–48]. It has been widely used for studying behaviour change, including for promoting adherence behaviour [48–50]. The framework details three main dimensions that drives behaviour change; Capability, Opportunity, and Motivation, each with a subset of behaviour change strategies. COM-B states that an individual needs adequate capability, opportunity, and motivation for a behaviour to take place (such as taking medicine), and that a deficit in any of these three areas means the behaviour is unlikely to occur. An individual’s capability to adhere to medicine and treatment may be affected by psychological factors of knowledge and memory; opportunity for adherence may be affected by access to medicine and support, as well as by physical and social barriers, and motivation may be affected by psychological factors such a self-confidence, values, and beliefs [48]. The COM-B framework provided theoretical guidance for developing the brief-text messages for the StAR2D intervention; the messages aimed to encapsulate key behaviour change strategies within the three domains (capability, opportunity, motivation) of the framework [38]. In this process evaluation, we continued our use of the COM-B framework, to assist our analysis of underlying reasons for participant responses [48].

### Data collection and analysis

Interviews and focus groups were conducted in private spaces, either at the health facility, or at the research agency (Lilongwe), or a nearby community center (Cape Town). For pre-trial interviews, using a semi-structured topic guide, the researcher explored experiences and perceptions of diabetes, sources of information and support, challenges, and opportunities in managing diabetes, experiences of receiving healthcare, access to mobile phones, attitudes to receiving health messaging via their mobile phones, as well as understanding and expectations of participation in the study (See Additional file 1 for the pre-trial interview guide). We analysed, but do not report separately on the pre-trial interviews. Pre-trial interview data helped with tracking the patient journey over time, and provide contextual information to better their responses to the intervention. In the post-trial interviews and focus groups, we focused on participants’ experience and perception of the brief messaging intervention, changes in adherence behaviour and health status over time, and whether text messaging may have influenced behaviour. (See Additional file 2 for the post-trial interview guide).

End of trial interviews happened once the participants had completed the trial and at this stage the qualitative researchers were given confidential access to the allocation status of participants. Training took place to avoid unblinding trial staff to individual allocation status. Data was captured with digital voice recording and was transcribed with anonymization. In Malawi, data collection was conducted in Chichewa, and translated into English during the transcription process by trained researchers (HN, CM, MN). In Cape Town, an English-speaking researcher (SC) conducted pre-trial data collection (most participants are bilingual), with the help of a Xhosa-speaking translator for the focus groups. In Cape Town, the post-trial interviews and focus groups were conducted by a researcher (NS), who is bilingual (isiXhosa and English). Interviewers were female, trained, graduate and post-graduate social scientists. Individual interviews lasted 45–90 min and focus groups lasted 90–120 min.

Interview summaries, capturing the key issues and researcher reflections, were completed within 36 h of conducting the interview, and was used for initial and iterative analysis of the data. The in-country lead researchers did this initial analysis together with the interviewer. Final analysis, based on interview summaries and transcripts was done by the first author (NL1). We used a combination of inductive and deductive analysis, using a coding framework informed by the categories explored in the interview schedule and by iterative analysis of interview summaries. We analysed the data along two dimensions. The primary dimension was an across-case analysis, where we compared and contrasted views across individual post-trial interviews, and between intervention and control group participants. A secondary dimension was analyzing individual pre-and post-trial interviews as pairs, for a longitudinal view of participant’s experiences and views. For the latter, we only report on notable changes from baseline interviews and contextual information for understanding an individual’s post-trial responses. Themes identified in FGDs supplemented the data from the interviews and allowed for data triangulation. Standard approaches to ensuring the quality of the methodology were used, including the use of a coding framework, confirmation of accuracy of initial analysis by the interviewers, reaching data saturation and reporting of divergent views.

Details of data sources and gender of participants are shown in Table 1 in the results section. In sum, a total of 28 individuals participated in individual interviews, with 26 of those being repeat interviews (pre-and post-trial with the same individuals) and 2 being only post-trial interviews (54 individual interviews in total). Twenty-five individuals participated in the 4 post-trial focus groups we conducted (2 each per country). The gender distribution was close to even for both the interviews and focus groups, with 26 females and 28 males participating overall. The age range of participants was 38 to 72 years and the duration of them living with diabetes (by the end of the trial), ranged from 18 months to 11 years. The level of co-morbidity was high, with most participants receiving treatment for at least one other condition, the most common being high blood pressure (others were high cholesterol, heart problems, asthma, HIV, anxiety, and depression).

### Ethical approval

Ethical approval to conduct the study was obtained from the University of Cape Town (126/2015), National Health Sciences Research Committee of Malawi (15/7/1425) and Oxford Tropical Research Ethics Committee (22–15). All participants who were included in the study provided written, informed consent to participate.

## Results

### Demographics

Details of data sources and gender of participants are shown in Table 1 and summarised in the Methods section. The table details the allocation status (intervention or control group in the trial) of participants in this qualitative study. A total of 16 individuals interviewed had been in the trial intervention group, while all 25 participants in the focus groups had been in the intervention group.

### General experience of the brief text message intervention

#### Expectations, acceptability, and varying engagement with brief messages

At the start of the trial, participants appeared to have a clear understanding of and reasonable expectations regarding their participation in trial. They hoped to learn more about diabetes and welcomed the idea of receiving messages to support their adherence to diabetes treatment.

At the end of the trial, participants were positive about the intervention, finding it acceptable, and useful. Several reported an improvement in their health, in terms of lowered and stabilized blood sugar levels (from high levels they had reported at the start of the trial). However, participants’ level of engagement with the brief messages and their responses to the messages varied. Some reported that the messages helped with the improvement in their health and adherence behaviour, while others felt they had no real need for it (though it could benefit others). A participant recalled different messages he found useful:

“*Yes, the ones they normally send you says: ‘Have you drunk your medication today?’, or ‘It’s close to your appointment’, ‘Remember to stay hydrated’, ‘Stay away from sugary foods.’”* (Male, SA, FGD)

Those who reported an improvement in their health felt this was due mainly to their increased confidence in managing their disease, helped by a greater acceptance of the disease and by family support. For some, it was the reminder messages that helped them to refill and take their medications more regularly. Healthy lifestyle messages were valued as reminders of the importance of diet, exercise, and stress management, and for its practical advice. More generally, the messages provided increased confidence and self-responsibility for taking their medications. A few participants expressed disappointment that the messages merely reinforced what they knew already, and a few would have preferred two-way communication that allowed for direct consultation with health staff.

#### Relational elements of the brief text messaging intervention

Participants identified messages and how this influenced their adherence behaviour, but they also identified more subtle relational effects that related to their participation in the trial. There are two elements to the relational effect. The first is that participants had appreciation for the content of the messages, as well as for the experience of receiving the brief messages. Messages generated a sense of encouragement and support, and it felt as if they had a supportive relationship with the sender of the messages. Message content was useful, but receiving a message also felt like an act of caring. One participant described it this way:

*“I feel like there is someone out there who cares about me, who thinks about me. He sends me a message to tell me to take my medication. It's nice to have someone who sends messages.”* (Male, SA, FGD)

A participant, who had been living with diabetes for 4 years, expressed a similar sentiment about feeling cared for, as if someone sent her a prayer:

*“You know, it not about the message. You send a message to my phone; I take it as a prayer … knowing there is somebody that cares.”* (Female, SA, IDI)

A second element is that some participants, in both the intervention and control groups, felt encouraged and supported by merely participating in the trial study, irrespective of receiving regular messages. Participants showed appreciation for the positive interactions they had had with research staff during the study enrollment and exit procedures. They recalled feeling respected and listened to, could ask questions, and learn more about their condition, without feeling judged. One participant said she had a similar feeling of being listened to when she saw a psychiatrist for anxiety, while another felt she had a companion, whom she could *“*talk to in my head”. Some in the control group had similar feelings of being cared for, especially when receiving a ‘Happy Birthday’ message.

### Experience and responses to brief text messages in relation to health

#### Reminder messages as prompts for refilling medicine

Participants in both sites knew the importance of refilling their medication and reported that most of the time, they remembered their appointment dates, and could collect their medicines on schedule. Participants continued using the same reminder strategies they reported at the start of study, such as checking their appointment dates intermittently, and relying on family to remind them. The reminder messages helped some participants play a more active role in developing strategies to remember, and to be less reliant on family. For instance, checking their due date in their patient-held record (called Health passport in Malawi) or their clinic appointment card (South Africa). Several participants acknowledged that they sometimes struggled to remember their medicine pick-up date (due to being forgetful, or a busy lifestyle), and some found the reminder messages particularly helpful.

*“I told you that I easily forget things, but the messages were reminding me, especially the clinic day, I tend to forget it, but the messages were reminding me.”* (Female, MLW, FGD)

The reminders were useful for the extra prompt it provided, even for those who considered themselves to be strict about their adherence, as expressed by one participant:

“*The SMS you guys sent us is very great. It helps because it encourages you to stay on this programme, and without this programme, you know, we are human, you will find ways of dodging the issue, preventing yourself from going to the doctor.”* (Male, SA, FGD)

Participants also spoke of a range of obstacles that sometimes prevented them from refilling their medication. In Malawi, these included economic factors, such as having no money for transport for their 3-monthly visits (some travelled for a 2-h journey by public transport) and having no money to purchase their medication privately when the clinic was out of stock. In South Africa, refilling required more frequent visits (monthly or 2 monthly). Due to clinic constraints, this usually required getting in line in the early morning hours before the clinic door opened, followed by long waiting times inside the clinic, clinic congestion and poor interactions with staff, experiences which made them reluctant to visit the clinic on their appointed dates.

#### Reminders to prompt and motivate taking medicine as prescribed

Participants indicated that for the most part, they take their medicine as prescribed, though several acknowledged it was an ongoing struggle. They continued to use the same reminder strategies as before the study, such as visual cues and daily routine, placing the medication on the bedside or kitchen table so its visible in morning, evening, and mealtimes. They also depended on family to remind them.

A few reported being strict about sticking to their medicine regimen (especially if also taking insulin) and having a sense of pride about being adherent to treatment. Some acknowledged not always taking their medication as prescribed, due to forgetfulness, or being too busy or tired. For them, reminder messages acted as a prompt to take their medications and reinforced the importance of taking medications regularly. A female participant who had been living with diabetes for 5 years (oral medication and insulin), said the messages helped her to become more self-reliant:

“*I no longer have this thing where you will find my sugar high. It’s all thanks to the SMSes, they help me. ‘Don’t forget your time’, … ‘Don’t forget your injection time’, ‘Don’t forget time to take pills.’”* (Female, SA, IDI)

Similarly, a female participant from Malawi, living with diabetes for 5 years, explained that the messages helped her to keep going with taking her medications despite the side-effects, whereas before she would miss dosages when she felt nauseas.

Even those with good adherence described having the occasional slip-up. A participant described how a perfectly timed reminder message once prevented her skipping her night-time dose:

*“I see they encourage us a lot, like this other day I was coming from my business, and I was very tired, and I was just dreaming of getting home, taking a bath and sleep. But before I slept, I just received the text asking me if I have taken my medications. That day I completely forgot; thanks to the message it reminded me, otherwise I could have slept without taking the medications.”* (Female, MLW, FGD).

A few participants also appreciated the messages about managing one’s medication in risky situations, such as social gatherings and travelling. They made sure to always keep a spare dose of medicine when travelling or attending social events.

Participants appreciated the importance of taking medicines regularly and long-term, but in many cases, despite their best efforts, their adherence behaviour fell short. They described a range of partial adherence beliefs and practice that had become part of their normal routine. The brief messaging did not seem to shift these beliefs and practices. Such behaviours included, for example, skipping one or more of their dosages on a regular basis, dropping one of their prescribed diabetes medications, taking a break from taking their medicine for a couple of days or even months, or substituting with alternative remedies. The partial adherence behaviours was reportedly in response to difficult and distressing side effects of diabetes medicine that are ignored by clinicians, and insufficient knowledge and counselling to understand why changes are made to their treatment. Other reasons include pill fatigue, beliefs about giving the body “a break” or “cleaning” the body from time to time, valuing alternative herbal remedies, concerns about different chronic medications “clashing” with each other, and suspicion of prescribed medicine with a different appearance. These beliefs and practices seem to be fairly entrenched for some, and often operated in parallel (even in contradiction) to their beliefs in the importance of following doctor’s orders.

#### Healthy lifestyle messages provide knowledge and motivation for healthy behaviour

##### Healthy eating

Participants were generally aware of the need to have a healthy diet, but reported a lack of adequate, useful, and timely information to guide them. They had concerns about what constitutes a healthy diet, and what local foods were available and affordable for people with diabetes. In addition to this knowledge deficit, participants had different levels of belief in the value of health eating, alongside medication. Some participants became more convinced over the years, while others became less convinced about the importance of health eating. A woman with substantial weight loss due to healthier eating (she dropped 3 dress sizes) became disillusioned about healthy eating when her blood sugar level did not stabilize. By contrast, a male participant who had a dramatic improvement in his health became convinced it was due to his running and weight loss. Malawian participants who did home-farming felt it kept them physically active and provided healthy produce for their families.

Participants noted that messages about healthy eating increased their awareness, made them conscious of what they are eating, reinforced their own ongoing efforts, and provided them encouragement and practical advice on what foods to eat and to avoid. They recalled messages on limiting fat, oily and sugary foods, and about using healthier food substitutes. A participant explained how these messages helped her manage her diet:

*“I remember that it is important to reduce fats and reduce sugar. If for example, I crave red meat, I need to ensure that I remove the fats, and to boil it instead of frying, so to assist the medication I take. It will be pointless for me to take pills and eat unhealthy because it might look like the medication does not work.”* (Female, SA, IDI)

A male participant who was diagnosed with diabetes the year prior to enrolling in the trial, said he was well-informed about what foods to eat and to avoid, but that the messages were useful as it helped him to better manage his cravings for the wrong foods. The messages also helped with managing household challenges of cooking and eating, such as getting family support for changing to healthier food options. A woman living with diabetes for 6 years, described how the messages helped to persuade her family to use healthier food preparation:

*“I was telling me children, please just boil my food, they refuse. They say I am just being stubborn. So, when I received a message from StAR2D talking about fried foods, I showed them and now they are still applying this knowledge whenever they are cooking, and they cook without adding oil”.* (Female, MLW, FGD)

Adhering to healthy eating at social events can be challenging and for a couple of participants, the messages helped to remind them to eat in moderation at social events.

##### Physical activity

Participants noted that although they were aware of the importance of regular exercise, they felt exercise was hard to achieve. They appreciated the messages for suggesting more feasible ways of keeping physical active. The exercise related messages motivated a few participants to increase their physical activity by walking more, doing more housework and gardening, and even considering dancing for exercise:

*“Yes, I started doing exercises instead of just staying [inactive]. And they even told us if you cannot manage to do exercises, you can put music on your phone or switch on the radio and start dancing.”* (Female, MLW, IDI)

##### Stress management

Some participants were convinced that stress was the reason for their high and fluctuating blood sugar levels, and some found the stress management messages particularly helpful. They felt stressed when their efforts to be adherent to treatment (taking medicine and healthy eating), did not lead to improvement in their blood sugar levels. Other stressors included loss of a spouse or other family members, and chronic worries about a range of issues (for example, ongoing ill health, one’s diet, substance abuse in the family, financial insecurity, visiting an overcrowded clinic and being scolded by clinic staff). The messages reminded and prompted them to make a conscious effort to better manage their stress. For example, avoiding getting stressed by other people’s problems, keeping busy as way of managing stress (one women helped in the family home shop), and reminding themselves to relax when their sugar level was high.

### Complex factors influencing diabetes adherence and engagement with the intervention

As mentioned earlier, participants reported a range of partial adherence behaviours that seem to be routine and influenced by individual beliefs as well as social factors and health service challenges. Partial adherence behaviour included reducing and skipping medication, taking alternative medicine, and struggles with eating a healthy diet, exercising, and managing their stress. Below we detail some of the underlying reasons.

#### Diabetes distress

The overall impression, from some participants, was that living with diabetes was difficult and distressing, more so than the other chronic diseases they suffered from (like hypertension, high cholesterol). The main reasons for the distress relate to the seriousness and discomfort of the symptoms, the fluctuations in blood sugar levels, unpredictability of health changes, and a chronic sense of not being able to control the disease, no matter their efforts. Living with co-morbid diseases further complicated the experience. Dealing with fluctuations of symptoms from multiple diseases was frustrating, as was the different intervals required for taking diabetes medication (compared to medicine intervals for their other chronic diseases).

#### Navigating complex and contested interactions with health services

Participants had an appreciation for efforts by the health services but found patient-provider relationships to need improvement. They felt their views were not sufficiently considered, that complaints of side effect were ignored, there was insufficient clinical communication about treatment, lack of affirmation of patient efforts, and in places, poor organisation of patient care and limited access to medicines – factors that contributed to stress and demotivation with managing their diabetes treatment.

#### What helps to manage diabetes?

Accepting one’s diagnosis and coming to terms with the need for lifelong medicine was a key factor in improving managing of diabetes. Acceptance meant worrying less about the underlying reasons for one’s condition, feeling empowered to take more self-responsibility for one’s treatment and health, and finding it easier to follow medical advice about adherence. A female participant living with diabetes for over a decade learnt from a ‘role-model’ diabetes patient in the community, who helped her to be more accepting of the illness, and to “… *just abide what we are told at the hospital”* (Female, MLW, IDI). Other sources of support came from family, friends, religious faith, ‘good diabetes’ patient role models, regular health awareness talks (mainly in Malawi), and counselling and affirmation from health care staff.

## Discussion

This process evaluation examined the experiences and perceptions of participants in randomised controlled study that tested the effect of brief text messaging support for adherence to diabetes treatment. It found that the messages were considered acceptable and useful, and improved their adherence behaviour for some. However, against a background of complex coping challenges, and the routine practice of partial adherence behaviours, their ability to act on the messages was limited. This context may explain in part, why the intervention did not have a positive impact on glycemic control. Process evaluations alongside trials are valuable for generating insights about the potential underlying causal mechanisms of the intervention and can inform development of other programmes [33].

### Similarities between Malawi and south African responses

Despite the differences in the way people live and access health services in Malawi and South Africa, the experience and perceptions of the text-message intervention was similar across sites. Malawi patients were a mix of urban and rural, had longer commutes, with 3 monthly visits, breaks in continuity of access to free diabetes medicine, and seemed to enjoy a more positive and trusting relationship with the health services. The South African patients were urban, living near the health facility, with monthly or 2 monthly visits, continuous access to free diabetes medications, but with more negative perception and less trust in the health service.

Similar complexities of living with diabetes emerged in both sites in the StAR2D intervention development study [45], as well as in other studies [11, 13, 14, 51, 52]. It may be that the complex influences that limit diabetes adherence, would also limit the impact of text messaging on adherence behaviour outcomes.

### Engagement and relational responses

In this study, those who expressed a benefit, indicated the reminder messages helped to prompt the desired behaviour (mainly in terms of jogging their memory), which speaks to the behaviour change domain of enhanced psychological ‘Capability’ in the COM-B framework. The reminders also reinforced their beliefs about the need for treatment and increased their sense of support and self-efficacy. This had the reported effect of motivating them to be more self-reliant and improve their adherence behavior, which speaks to the ‘Motivation’ domain of COM-B framework. Nevertheless, for the most part, the messages did not seem to change their beliefs and practices around partial adherence, for example, taking treatment breaks and use of alternative remedies. There was also limits to the ‘Opportunity’ domain, as the messages did not make a difference to the physical or social challenges shaping adherence behaviour, such as the complexity of the medical regime, and challenges around accessibility and experience of healthcare services. The barriers to the Motivation and Opportunity domains of the COM-B framework may in part explain the limited adherence behaviour change observed in the trial outcomes.

Studies have found that while text messaging often has wide acceptability, with positive effects in some areas, like smoking cessation and antiretroviral medication adherence [17], the evidence for diabetes is mixed [20, 21, 24, 25, 53, 54]. In a study showing long-term success for diabetes management, the intervention was multi-modal; tailored and interactive text messaging, self-monitoring relating to use of insulin, and ability to contact a health care provider [22, 23, 55].

A cross-cutting issue is the relational aspect of the mHealth intervention, which had two elements. Firstly, participants in both settings appear to value not only the content of the text messages, but also the sense of support and encouragement they got from merely receiving messages; almost as if they had established a virtual caring relationship with the sender of the message. Secondly, they also experienced a sense of support and encouragement from participating in the research study, whether they were receiving the intervention or not. This came from positive interactions with research staff at the beginning and end of the study. These relational effects were more pronounced for South African participants; perhaps as it contrasted more starkly with their negative perceptions of the health services. A similar relational effect of digital messaging was found in the Cape Town hypertension adherence study [12], in Kenya with anti-retroviral adherence [56] and in a high income country setting, with cardiovascular adherence support [57].

### The complexities of adherence to diabetes treatment

The study highlighted a range of complex issues that interact with and contribute to routine practice of partial adherence behaviours (for medical and lifestyle adherence). Participant responses shared a sense of lack of control over, and limited capacity for improving their diabetes-related health. The challenges relate to knowledge gaps on how to turn information into action, gaps in continuous access to medicine, complexity of the medical regime, healthcare interactions that are demotivating, instability in symptoms of diabetes, debilitating side effects that are not addressed, and beliefs and practices that negatively shape their adherence behaviour. Living with other co-morbid disease further complicated their efforts to manage their treatment regimens and to maintain good health. Individual, social and health service complexities such as those found in this study, has also been reported across settings and patient groups, prompting calls for more innovative, multi-dimensional, and tailored adherence interventions [11, 15, 25, 51, 58–60].

It may be difficult for a text message by itself, to be effective in improving adherence behaviour and clinical outcomes, against a background of an overburdened health system, and where partial adherence beliefs and practices are entrenched. While participants acknowledged support efforts by health services, they experienced health care as not sufficiently responsive to their informational, clinical and support needs. A systematic review of trials in other African settings, using one-way brief text messaging confirmed the uncertain clinical effects; with no clear evidence of SMSes improving diabetes and hypertension management [53].

We used automated unidirectional SMSes to improve the feasibility and scalability of the intervention, but lack of direct, personalised communication with health care workers may have contributed to the limited intervention impact. A global review of tailored text messaging for improving diabetes management reported moderate improvements on blood glucose levels, but mostly in high-income countries [60]. The review suggested that certain aspects of tailoring messages may work better, such as non-automated, direct, and personalised communication. For example, where messages are sent from a real health care worker, and when SMS communication is combined with other assessment tools. The directionality (unidirectional versus bidirectional automated messaging) did not directly impact the effectiveness of the interventions [20, 60].

### Implication of the findings for research and practice

In the COM-B behaviour change model, all three of the components of capability, motivation, and opportunity, are needed for positive behaviour change to occur [46, 47]. Text messaging seem to address the capability and motivation components, as evidenced by reports of feeling encouraged and motivated, though messages could not change social and physical opportunity barriers to adherence. Complex, multi-dimensional interventions may need to go beyond informational and motivational support, to address the interactions of patient belief systems and practices and to improve trust and collaboration between providers and patients [25, 48, 59]. Testing of complex interventions would need to be balanced with the feasibility of implementing such interventions at scale.

### Strengths and limitations of the study

The process evaluation was part of a comprehensive evaluation which included evidence- and theory-informed intervention development and a costing study, alongside a pragmatic randomised trial [39]. The longitudinal perspective (interviewing the same individuals pre- and post-trial) provided useful contextual information and enriched our understanding of participant’s experiences and views over time. Triangulating the use of individual interviews and focus groups helped to strengthen the credibility and reliability of the findings and the sample had good representation from both genders. We think the study was strengthened by use of mother-tongue interviewers that allowed for better understanding of cultural nuances, and where this was not feasible, we used translation support. The lead researchers of the qualitative study (NL1 and HM) were also co-investigators on the main StAR2D trial, and we ensured that there was an appropriate level of independence between the qualitative and quantitative research team. We planned to interview a greater ratio of individual participants in the intervention compared to control group, but this was a challenge due to logistics of keeping allocation status confidential during the trial. We would have liked to arrive at a more in-depth analysis of how the COM-B behaviour change strategies operated, but we think this requires a combination of methodologies including use of traditional qualitative approaches, together with more focused, innovative qualitative techniques, such as cognitive interviewing (used for market research), and perhaps patient diaries. Combining this with quantitative data on implementation processes outcomes (coverage, fidelity), on quality of the health services, and stakeholder views, may provide more comprehensive insights to explain the impact of experimental studies [31, 36, 46].

## Conclusion

This process evaluation study provided context and insight into the factors influencing participants’ responses to the text messaging intervention in a trial aimed at improving diabetes clinical outcomes and adherence behaviours. While participants found brief text messaging acceptable and useful, the complexity of the context in which patients take their diabetes medication, may explain in part, why brief text messaging was insufficient to improve clinical and behaviour impact of the trial. The scale of need for self-management and health service support suggests that digital approaches alone may be unsuccessful, unless accompanied by health system strengthening and other forms of self-management support.

## Supplementary Information

**Additional file 1.** StAR2D pre-trial interview guide. This the interview guide used for individual interviews and focus groups with participants at the start of the trial.

**Additional file 2.** StAR2D post-trial interview guide. This is the interview guide used for individual interviews and focus groups with participants at the end of the trial.

## Data Availability

The datasets that support the findings of this study are available from Dr. Natalie Leon (author NL1) Restrictions apply to the availability of these data, which were used under license for the current study. Reasonable request for data access will be considered by Dr. Leon and the PI of the StAR2D study, Prof Andrew Farmer.

## Abbreviations

COM-B: Capability, opportunity, motivation - behaviour
FGDs: Focus groups discussions
IDIs: In-depth interviews
LMICs: Low-and middle-income countries
MLW: Malawi
SMS: Short message service
StAR2D: SMS text adherence suppoRt for people with type 2 diabetes
South Africa: SA
T2DM: Type 2 diabetes mellitus
