# Supplementary material for: Process evaluation of a brief messaging intervention to improve diabetes treatment adherence in sub-Saharan Africa
Source: BMC Public Health. 2021 Aug 21;21:1576. doi: 10.1186/s12889-021-11552-8 (PMC8379852; doi:10.1186/s12889-021-11552-8)
Supplement: Supplementary file 1 — Additional file 1. StAR2D pre-trial interview guide. This the interview guide used for individual interviews and focus groups with participants at the start of the trial. [file 12889_2021_11552_MOESM1_ESM.docx]

**StAR2D Patient Interview Guide: Process Evaluation (Pre-trial)**

**Welcome/introduction**

Thank you for agreeing to take part in this study. Before I begin I would like to explain why we would like to talk to you. You have recently signed-up to participate in the *StAR2D study. This study is investigating whether using SMS text-messages to keep in contact with people who have sugar diabetes (and who are on treatment) makes a difference to blood sugar control. The *StAR2D study is being organised jointly by the Medical Research Council in the United Kingdom and Global Alliance for Chronic Disease. We are doing an Interview study alongside the StAR trial study to find out more about what is required to implement the *StAR2D study and what is the experience of all the participants, including patients who participate in StAR, staff and managers and the StAR researchers.

For this interview, we want to hear your views and experiences of living with diabetes to better understand your experience of living with a long- term disease and your experience of your health care. We would also like to understand how you currently use your mobile phone and what you are expecting from participating in the *StAR2D study. All of this will help us better understand the opinions and experiences of the people who are participating in the *StAR2D study.

It is important to emphasise that there are no right or wrong answers. We want to hear about your views and experiences from your own perspective. Also, if you feel uncomfortable about answering any of the questions, you do not have to answer them

This interview will take between 45 and 90 minutes. If you agreed, we will record our conversation, but the recording will only be heard by members of the research team. When we report our findings, any of your words/quotes will not have your real name attached, so no statement could be linked directly attributed to you. This study is for the purposes of research only and aims to contribute new information that could help improve adherence to diabetes treatment.

Do you have any questions before we start? If not, you can ask anytime while we are talking and again at the end of the interview. Go through the information on the consent form with the participant. And ask participant to complete and sign the consent form if they are still happy to be interviewed.

Fill out the demographic detail form.

**Questions**

**Demographic information**

1. Can you please tell me a little bit about yourself?

a) Where do you live?

b) How long have you lived here?

c) Who do you live with?

d) Do you spend time away visiting other areas in South Africa/Malawi (for work/study/visiting family)?

**General experiences and perceptions of diabetes**

2. When were you first diagnosed with diabetes?

a) When you were first diagnosed, how did you feel?

b) Do you still feel the same way or differently? If differently, what changed?

**3. What is your understanding of diabetes as a disease?**

a) What do you think causes diabetes?

b) How do you think diabetes can best be managed?

**4. What is your experience of living with diabetes at the moment?**

a) What are some of your biggest challenges with managing diabetes?

b) What has worked well for you in managing/coping with diabetes?

c) How confident are you currently in managing your diabetes?

**5. Have you told any people who are close to you about your illness e.g. partner, other family members, friends, neighbour?**

a) If so, how did they react? If not, can you explain why you haven’t told anyone close to you?

b) If applicable: Do you feel the people you have told support you?

c) Are there any other people or places which provide you with support?

**6. Where or from whom do you receive information/advice about diabetes?**

a) Do you find this information/advice helpful?

b) Whose advice are you most likely to follow? Why?

**General experiences and perceptions of taking and adhering to medication**

**7. Can you please tell me about your experiences of taking medication for your diabetes?**

a) What medication do you take? How often do you take it? (Check if person names medication and dosages. If not, check what they do know).

b) How do you feel about taking this medication?

c) Many people find it difficult to take all their tablets and occasionally miss one or two. Is that a problem for you? If so, why do you think this sometimes happens for you? Can you describe a recent incident when you forgot to take your medication?

d) How do you remind yourself to take your medication i.e. any reminder strategies? Please tell me more about these and how well they work for you.

e) Do you get any support from others to help you with taking your medication?

f) Do you take medication for other diseases? If so, which ones? Does this interfere with taking your diabetes medication?

**If participant does not raise anything themselves about side-effects:**

g) Some people complain about side effects of their medication. What has been your experience? If they have experienced side effects: How has it affected your taking of meds in the short-term and longer term? What have you done about this?

**General experiences and perceptions of receiving health care at the clinic**

**8. Can you tell me about your experiences of receiving health care at the clinic? (avoid a lengthy description of broader aspects of treatment – just enough to understand – and focus on the following areas, prompting as necessary)**

a) How long have you been getting treatment at [name of clinic]?

b) How often do you go to [name of clinic] for check-ups/collecting drugs/other? Do you ever attend a different clinic?

c) How do you get to [name of clinic]?

d) Can you talk through all the steps involved when you come to [name of clinic]? For example, how do you get an appointment? When you come how long do you usually travel for, wait before being seen at reception, the nurse, the doctor, the pharmacy?

e) How often do you see the doctor?

f) Do you go to the ‘club’? If so, how often? If so, what do they do at the club? And how is that working for you?

g) What makes getting care from [name of clinic] easier/harder? What, if any, are the main challenges you face at [name of clinic]? What, if any, do you think works well at [name of clinic]?

h) Does going to [name of clinic] interfere with doing other things? If so, how/why? What would work better?

**9. I’d now like to hear about your experiences of picking-up your medication at [name of clinic]:**

a) How often do you collect your diabetes medication from [name of clinic]? Do you ever collect your diabetes medication from a different clinic?

b) How do you go about collecting your diabetes medication from [name of clinic]? Do you collect your medication yourself? Do you have any problems in doing this?

c) If someone else collects your meds, could you tell me more about how this works and the benefits/problems?

d) How do you remember to collect your medication? I.e. do you have a system for remembering/reminder system? If so, how well does this system work?

e) Does it sometimes happen that you forget to pick up your medication from the clinic? If so, how often does that happen? Why do you think this sometimes happens? Can you describe a recent incident when this occurred?

f) What happens when you forget to pick up your meds? How soon are you able to pick up the batch you missed?

g) Do you ever run out of medication? If so, what do you do when you run out?

h) Are there times when you do not take any meds/your diabetic meds for a while? How often in the last year did that happen? When last did that happen? How long were you without meds on that occasion? How did you feel/manage your health then?

**Cell phone usage**

**10. Do you own a phone? Do you have more than 1 sim card? If yes, when/why do you switch between the two? (Did you inform the *StAR study co-ordinator about this – so as to help them stay in touch?)**

**11. Do you feel comfortable using your phone? Do you perform most functions yourself, or do you usually get someone to help you? (I.e. try to get an impression of familiarity and ease of use) Get them to show you the different functions they use mostly- e.g sms, What’sApp, voice/sms contact list etc.**

**12. How often do you use your phone? What do you mainly use your phone for? (E.g. Calls? Messages?- explore more; Reminders?- explore more , Calendar?- explore more; Calculator? Flash light? Radio? Web access? Facebook? WhatsApp?)**

**13. What kinds of SMS-ses do you usually get? Do you ever receive SMSes besides from friends and family (e.g. from Bank, Eskom, Clinic, Adverts)? If so, how do you feel about receiving these kinds of messages? In what language do you usually receive SMS-ses? In what language do you usually respond to SMS-ses/ Whats’App, Voice calls?**

**14. How often is your phone switched on and with you? When do you switch it off? Do you ever lend your phone to someone (e.g. your partner, a friend, a relative)?**

**15. Do you ever experience any problems with using your phone (e.g. no signal, out of battery, unsafe or inappropriate to use?)**

**16. Does your phone ever get lost? Or stolen? If so, how often? When this happens, how long does it take you to get another phone? When you get a new phone, do you usually get a new number or keep your old number?**

**Expectations of StAR trial**

**17. What are your initial thoughts/expectations about the *StAR trial?**

a. Why did you decide to sign-up for the study?

b. What is your understanding of the aim of the study?

c. And what do you know about what will happen next in this study, and in the next 12 months?

d. What do you hope to gain from participating in the study?

e. Do you think the SMSes you get from the *StAR2D study will fit/help with the reminder systems you currently have for taking your meds and collecting them from the clinic?

f. Do you have any concerns or worries about participating?

g. Do you foresee any problems with the study?

**We are now wrapping up the discussion**

**a) Do you have any final comments or questions?**

b) Thank you so much for your time and your willingness to share your views and experiences.

c) Can I check that you are still happy for us to use what you have told us in our research?

d) At the end of the research we will be once again carrying out interviews with people who took part in the *StAR2D study. Would you be interested in taking part in another interview at this point? If yes, are you happy for us to contact you on your cell phone?

**Handing over reimbursements, patient and researcher sign the reimbursement form**.
