# Supplementary material for: Process evaluation of a brief messaging intervention to improve diabetes treatment adherence in sub-Saharan Africa
Source: BMC Public Health. 2021 Aug 21;21:1576. doi: 10.1186/s12889-021-11552-8 (PMC8379852; doi:10.1186/s12889-021-11552-8)

**PARTICIPANT INTERVIEW MASTER GUIDE: STAR2D PROCESS EVALUATION (POST-TRIAL)**

*StAR2D

**WELCOME/INTRODUCTION**

***Please complete all the Informed consent procedures prior to the start of the Interview questions.***

Thank you for agreeing to this interview. (Researcher name), a researcher from the Malawi Epidemiology Intervention Research Unit (MEIRU)/ South African Medical Research Council (SAMRC), interviewed you about a year ago, just before you started the StAR2D Trial. I am also from MEIRU/SAMRC and am now doing a follow-up interview.

- This interview is different from the one you did with the StAR2D research team that enrolled you.
- For this interview, we would like to know a little more about your experience and opinions about getting SMS text-messages over the past year. This will help us better understand the opinions and experiences of the people who are participating in the *StAR2D study.
- It is important to emphasise that there are no right or wrong answers. The Interview team is a separate team from the trial team and we want to hear about your experiences from your point of view, to understand also how we can improve this kind of intervention. Also, if you feel uncomfortable about answering any of the questions, you do not have to answer them
- This interview will take between 60 and 90 minutes. The recording of the interview will only be heard by members of the research team and we will not use your real name when we write about this. This study is for research only and will bring new knowledge about how to help people with their diabetes treatment
- **Do you have any questions before we start? If not, you can ask anytime while we are talking and again at the end of the interview.**

## QUESTIONS

**DEMOGRAPHIC INFORMATION**

Please familiarise yourself with this information from the pre-trial interview, as part of the preparation for the post-trial interview. Briefly check the information if not all the information is available.

***Gender:***

***Age:***

***Duration of diabetes:***

***Educational background: (primary, high, tertiary)***

***Employment status:***

***Job name:***

1. **GENERAL: LOOKING BACK: EXPERIENCES AND PERCEPTIONS OF DIABETES**

Review of the past year since the last interview:

- Update on general well-being & health status; explore any changes and reasons (listen out for possible hooks for more specific questions on the trial experience noted below)
- Establish to what extent the participant felt that remembering to pick-up meds, taking meds as prescribed, general awareness of diabetes disease and health living were areas they identified then (and now) as needing help with.
- Establish what areas the participant previously (in baseline interview- see their Interview summary) identified as their main areas of concern regarding living with diabetes.?
- **Keep this background information in mind during the interview – so as to personalise the questions more, especially regarding influence of SMS-ses on behaviour.**

1. ***I’d like us to look back over the last year since you started with the StSR2D trial. What was the past year like for you in terms of your general health?***
2. ***What is your experience of living with diabetes at the moment?***
3. ***You mentioned before a main challenge for you was: X (see previous interview).***
   1. ***Is this still the case and how have you been managing this lately?***
4. ***What has worked well for you in managing/coping with diabetes this past year?***
5. ***How confident are you currently in managing your diabetes?***
6. ***Was there any time this past year when you did not have meds? How did this happen and how did you handle it?***
7. ***Any struggles with taking your meds according to the way the doctor prescribed it? Any time this year that you skipped taking your meds for any reason? Why was that and how did you handle it?***
8. ***Any struggles with how you experience the treatment at the clinic this past year?***
9. **EXPERIENCE/ PERCEPTION OF PARTICIPATION IN THE TRIAL**

Open ended reflection on what it was like to be part of the trial. Reflection on what the experience was like. Reflections on experience over the past year.

Spend limited time on this as we will ask more detailed questions on response to SMS messages later- use it as a bridge to rest of interview questions. (*Limit the questions if the person was in the Control group and received mainly trial related text messages.)*

1. ***What was the StAR2D trial all about for you?***
2. ***What stood out for you?***
3. ***What worked well/ Not so well ?***
4. ***What was your ideas and expectations when you joined the trial?***
5. ***What expectations were met and what were not?***
6. ***What things would you have liked to be different/ see changed?***
7. ***Would you recommend the programme to other health facilities, why and why not?***
8. ***Do you think you are better off or worse off after the intervention, please explain?***

**3. CELL-PHONE USE & DELIVERY MECHANISMS OF THE SMS INTERVENTION**

Explore participant experience and perceptions of the appropriateness, acceptability, usefulness of the intervention delivery mechanisms with reference to areas below. Start with cell-phone related questions, then move to more open ended questions about the SMS intervention delivery.

**3.1 Cell-phone usage:**

1. ***Which phone did you use to get the SMS-ses messages?***
2. ***Check if they have the phone on them and if they can show you some of the messages on the phone ( if they kept any)***
3. ***In the past year, did you have to change phones or sim cards or your cell-phone number? Why is that and how did that affect the SMS messages from the StAR2D study?***
4. ***How did you access the SMS-messages on you phone? (Check if they needed help; accessed it by themselves or with help of others, and if there were any difficulties.)***
5. ***In the past year, have you ever been without your phone for while. How did that affect your StAR SMS messages?***
6. ***Did you experience any difficulties in being able to read the messages (perhaps due to eyesight etc.). How did you handle that?***
7. ***Was your phone ever switched off for a long period? How did it affect your SMS-messages?***

**3.2 SMS- intervention deliver mechanisms:**

Here we want to know about their experience and perceptions of the following delivery mechanisms (i-vi below): Use general probes a-c below. *(Limit the question for participants who were in the control group)*

1. ***What do you think of how the intervention was delivered in terms of (go through each of the items above)***

***b) What worked well/ not so well?***

***c) What things/aspects would you have liked to be different/ see changed***

1. Quantity/volume of messages
2. Frequency of delivery (3-4 messages per week)
3. Timing of message delivery
4. Overall duration of message delivery period
5. Language in which the SMS was delivered to you
6. One-way communication function (only receiving information; not able to text back or communicate with clinic personnel/ health care professional).

**4. CHANGES IN EXPERIENCE OF DIABETES AND LINKS TO STAR2D PARTICIPATIONS**

NOTE: This section makes a generalized attempt to explore if the participants identify any behaviour change (for the better or worse) in their adherence to fetching and taking meds, general healthy living and/or general sense of well-being. And if a change is reported, we need to:

- get a sense of what they think the reasons are for the change
- explore what if any connection they think the SMS-ses had to this change (this section is also explored further in No.5 later) (*Limit this question for those in the control group)*

1. ***We spoke earlier about how you have been living with diabetes and if you saw any changes. I’d like to ask more about that, if that’s okay. Would you say anything has changed with the way you are handling/managing/responding to your diabetes illness and treatment. Or would you say things have been pretty much the same?***
2. ***PROBE: Could you explain your answer a bit more please? What has changed/not changed? And what do you think the reasons are?***
3. ***How has your participation in the StAR study made a difference or not made much difference to the way you feel about your illness and about the clinic? Please elaborate (explore both difference in feelings about illness, self and about the clinic)***
4. ***PROMPTS (and explore examples and reasons)***

- Changes in behaviour around collection and taking of meds
- Changes in level of knowledge about the disease
- Changes in own attitude to illness, treatment and health living? (look for examples of increased sense of self-responsibility and self-efficacy)
- Changes in actual behaviour around health living interaction with the clinic
- Changes in attitude to, expectations of, and experience of the clinic and clinic staff

**5. RESPONSES TO MESSAGE CONTENT AND BEHAVIOUR CHANGE MECHANISMS**

**(CORE SECTION OF INTERVIEW)**

Examine the perception of participation of the SMS message content in terms of acceptability, usefulness, their responses to message content and influence on behaviour change. *(Limit these questions for those in the control group)*

NOTE: This is the area where we hope to explore in more depth, if (and how) there are any connections between reported behaviour change and responses to the SMSes. E.g. to see if participants can identify particular responses to SMS-ses in terms of their thoughts, emotions, actions AND if they give provide any indication that their changes in behaviour is connected somehow to receiving SMS-ses. So we want to explore as far as we can (without leading the patient’s answers), if the participant is able to describe What, How and Why a change occurred- and if any of those response can roughly be matched to the behaviour change strategies outlined in the Behaviour change taxonomy. We are looking for any indications that the messages elicited changes in the following areas:

- Capacity/Capability for change; psychological or physical ability to enact the behaviour
- Motivation/Support for changing behaviour; Reflective and automatic mechanisms that activate or inhibit behaviour
- Opportunities to change behaviour: Physical and social environment that enables the behaviour

*Note: Question g) below and the questions in section 5 are key questions to draw out some of the detail regarding possible underlying behaviour change strategies that were involved- e.g. issues like information, reminders/prompts, support, reinforcement, sense of own agency, self-efficacy, intention for change, readiness, capacity for & opportunity for change.*

1. ***How did you find the messages (and what it was trying to say? )***
2. ***Any messages that stood out for you and why?***
3. ***What was your favourite message and your least favourite message? Why?***
4. ***What do you think the messages were trying to achieve?***
5. ***Do you have any examples where you think the messages did achieve this effect with you?***
6. ***Are any particular type of messages that meant more to you than the other ones? (If so, why?)***
7. ***Any messages that you found particularly helpful or not helpful and why?)***
8. ***What usually happened when you receive messages? (Explore if they had a particular reaction in terms of their thoughts, emotions and actions/behaviour) .***

Note: The interviewer can spend more time exploring these questions and return to it at any stage of the interview if the opportunity arises/as appropriate.

1. ***If not mentioned, check:***
   1. ***Did you store messages on the phone and why? Do you want to show me some of the stored ones?***
   2. ***How about sharing the messages with others- was that something you wanted to do and why? Which SMS-ses would you have shared if you could? why***
2. ***Would you have preferred different messages? Or a different way of conveying some of the messages? (Please elaborate)***

NOTE: Probe: If no mention is made of different categories of messages- ask specifically;

***j) Do you remember any examples of messages that was about the following topics? And if so, which of these types of messages did you find more useful or less useful?***

1. reminding you about fetching your meds at the clinic
2. reminding you to take your meds as prescribed
3. general information about diabetes disease
4. general information about taking precaution to limit the complications of diabetes
5. encouragement about trying your best to take care of yourself and the disease
6. about exercise
7. about food
8. about managing stress
9. smoking and drinking
10. about seeking support from others
11. about talking with health care providers about your health concerns
12. **CONCLUSION AND RECOMMENDATIONS**

***We are coming to the end of the interview where you can also ask questions. First, I’d like to hear if you have any recommendations about this SMS project. (Adjust for those in the control group)***

1. ***If the clinic was to offer something similar -where they send out such SMS-ses as reminders and about how to take care of your health- would you recommend to family members or friends? Why/why not***
2. ***What advice would you give the clinic managers and staff about how to make such a SMS project work well?***
3. ***Do you have any other ideas about how such and intervention using SMS-ses can be used to help others in the health services? For example? Anything else you would want to add to the SMS-ses?***
4. ***The trial has now come to an end and you will not receive further messages from the StAR2D team. Do you understand what that means? (check if they know that they will not receive any more SMS-ses from StAR2D)? Any further comments?***
5. ***Do you have any questions you’d like me to answer?***

Thank you very much for your time and effort. We really appreciate it. Your opinions will help us to learn more about how using SMS-ses can help people to improve their health.

**Handing over reimbursements and participant and researcher signing for it.**


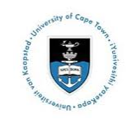

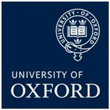

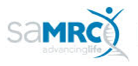

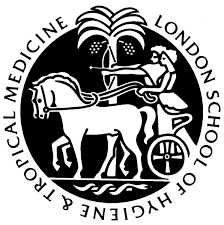

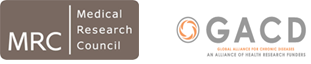

Supplement: Supplementary file 2 — Additional file 2. StAR2D post-trial interview guide. This is the interview guide used for individual interviews and focus groups with participants at the end of the trial. [file 12889_2021_11552_MOESM2_ESM.docx]
